# Sample size determination: A practical guide for health researchers

**DOI:** 10.1002/jgf2.600

**Published:** 2022-12-14

**Authors:** Alaa Althubaiti

**Affiliations:** ^1^ College of Medicine King Saud bin Abdulaziz University for Health Sciences Jeddah Saudi Arabia; ^2^ King Abdullah International Medical Research Centre Jeddah Saudi Arabia

**Keywords:** effect size, power, regression analysis, sample size, study design

## Abstract

Although sample size calculations play an essential role in health research, published research often fails to report sample size selection. This study aims to explain the importance of sample size calculation and to provide considerations for determining sample size in a simplified manner. Approaches to sample size calculation according to study design are presented with examples in health research. For sample size estimation, researchers need to (1) provide information regarding the statistical analysis to be applied, (2) determine acceptable precision levels, (3) decide on study power, (4) specify the confidence level, and (5) determine the magnitude of practical significance differences (effect size). Most importantly, research team members need to engage in an open and realistic dialog on the appropriateness of the calculated sample size for the research question(s), available data records, research timeline, and cost. This study aims to further inform researchers and health practitioners interested in quantitative research, so as to improve their knowledge of sample size calculation.

## Graphical abstract

This review aims to explain the importance of sample size calculation and to provide considerations for determining sample size in a simplified manner. Five elements essential in sample size calculation are discussed, and practical examples are used. This study aims to further inform researchers and health practitioners interested in quantitative research, so as to improve their knowledge of sample size calculation.

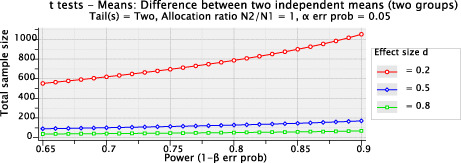

## 1 INTRODUCTION

In the initial stage of planning a research study, sample size calculation—or power calculation—answers the question, “How many participants or observations need to be included in this study?” If the sample size is low, the research outcome might not be reproducible.^1^ Informal guidelines for sample size based on the experience of researchers are used in most research studies and may be sufficient, as is the case in pilot studies.^2^, ^3^ However, when funding or institutional review board approval is requested, review committees often expect an explicit justification of the sample size. An increasing number of academic journals have requested evidence of sample size calculation or specific requirements to be provided in the method section of a manuscript, and the calculation can be part of a checklist before submission to a journal.^4^, ^5^ In addition, when sample size calculation is not mentioned, reviewers may wonder whether the sample size is adequate.

Despite the many instructional materials available on sample size calculation, as well as statistical background knowledge being increasingly common among practitioners,^6^ sample size calculation can be very challenging for researchers.^1^ In addition, although sample size calculation is requested as part of the method section of a manuscript, adding this requirement has not obviously increased the reporting of sample size.

The aim of this methods review is to present the importance of sample size calculation and to highlight factors worth considering when describing the rationale for the selected sample size. The different equations for sample sizes are not illustrated here, as they are beyond the scope of this review. Instead, challenges relating to sample size calculations in health research are summarized.

The remainder of this paper is organized as follows. In Section 2, some important terms are presented. Sections 3, 6 discuss sample size calculations according to various types of study designs. Finally, Section 7 offers some general recommendations.

## 2 SAMPLE SIZE: WHAT TO UNDERSTAND?

Sample size calculation involves several statistical terms, a selection of which is provided below in Table Table S1. In the following sections, the basic concepts are discussed, and detailed guidance is provided for sample size calculation.

### 2.1 Expectations regarding sample size

A sample size can be small, especially when investigating rare diseases or when the sampling technique is complicated and costly.^4^, ^7^ Most academic journals do not place limitations on sample sizes.^8^ However, an insufficiently small sample size makes it challenging to reproduce the results and may produce high false negatives, which in turn undermine the scientific impact of the research. On the other hand, choosing to enlarge the sample size may be ethically unacceptable, particularly in Phase 1 studies, where human subjects are exposed to risks. Moreover, a very large sample size may lead to *p*‐values less than the significance level even if the effect is not of practical or clinical importance (i.e., false positives).^9^ Hence, sample size calculation is important for striking a balance between risk and benefit.^10^ Researchers' focus should not be on producing large sample sizes. Instead, the focus should be on choosing an appropriately sized sample that achieves sufficient power so that statistical testing detects true positives, comprehensively reporting the analysis techniques and interpreting the results in terms of *p*‐values, effect size, and confidence intervals.^8^, ^11^

### 2.2 Sample size calculation using software programs

Sample size calculation need not be done manually, and there are several free‐of‐charge software tools that can assist in the calculation. For example, OpenEpi^12^ (an open‐source online calculator) and G*Power^13^ (a statistical software package) are commonly used for sample size calculations. Wang and Ji^14^ provide an online calculator for common studies in health research. PS Power and Sample Size Calculation^15^ or Sample Size Calculator^16^ are practical tools for power and sample size calculations in studies with dichotomous, continuous, or survival outcome measures. The support offered by these tools varies in terms of the type of interface and the mathematical formula or assumptions used for calculation.^17^, ^18^, ^19^, ^20^

### 2.3 Statistical analysis to be used is important in sample size calculation

Predominantly, the sample size should be determined based on statistical analysis.^2^, ^21^, ^22^ The type of analysis should be closely related to the study design, study objective, research question(s), or primary research outcome. Most sample size calculation software packages include the option to select the required statistical test related to the response or outcome variable(s), with each test requiring a different sample size. Therefore, if a comparison between two or more groups is required after estimating the frequency of a certain attribute in the population, the calculated sample size should be adjusted, in order to account for the types of statistical tests to be used in the comparison. This ensures that the final sample size is appropriately suited to the study's main objective(s) or hypotheses.

### 2.4 When possible, determine the effect size

In studies examining the effect of an intervention/exposure or the difference(s) between two or more groups, the effect size must first be determined, in order to calculate an appropriate sample size. The effect size is defined as the minimum effect an intervention must have in order to be considered clinically or practically significant.^23^ This is considered the most challenging step in sample size calculation. When the effect is small, identifying it and reaching an acceptable level of power requires a large sample. When the effect is large, it is easily identifiable; hence, a smaller sample size is sufficient.

The size effect is mostly determined by experience or judgment.^24^ It can also be estimated from previously implemented, well‐designed studies (such as meta‐analyze; see, for example, Thalheimer and Cook^25^ for a simplified illustration on how to determine effect size from published research). An initial pilot study may determine the effect size for start‐up studies if accompanied by conversations with experts in the field that provide useful information on adequate value for the effect size. In a pilot investigation, sample size calculation may not be required for the pilot sample.^26^ An important approach worth considering here involves enrolling pilot study participants based on the inclusion and exclusion criteria of the planned larger study and then testing the feasibility of the methods.^27^, ^28^

Various solutions have been proposed for cases where effect sizes cannot be determined. Cohen^29^ recommends using small, medium, and large effect sizes instead of specific values (i.e., standardized or unit‐free effect size). For example, when the mean difference between two groups is of interest, and independent samples t‐test is to be used, the standardized effect size is calculated as:

$$\text{Standardized effect size}=\frac{\text{difference between}\;\mathrm{two}\;\text{means}}{\text{standared deviation of response}}$$

The difference between the two means is the difference in practical importance, and the standard deviation of the response is often estimated from similar previous studies.

**FIGURE 1:**
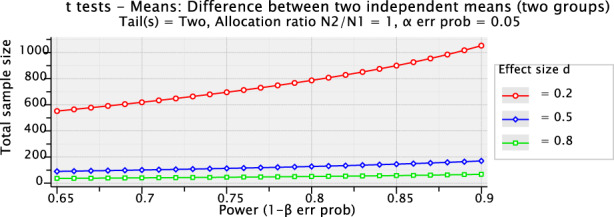
G*power sample size software is used. Tail(s) = two: Two‐tailed *t*‐test. Allocation ratio N2/N1: Intended relative number of participants in each of the comparison groups (e.g., 1 or 2). *α* err prob: The probability of type I error = 0.05. *β* err prob: The probability of type II error.

Figure 1 illustrates that a sample size can be based on a range of standardized effect sizes and powers (e.g., *d* = 0.2 [small], 0.5 [medium], or 0.8 [large]). If the aim is to compare the mean difference between two groups, and an effect size of 0.5 (medium) is used, the total sample size required to reach a power of 80% is 128 participants. Hence, 64 participants are included in each group.

By contrast, for a small effect, the total sample size required is 788 patients, with 394 patients in each group. The use of such arbitrary values is common in sample size calculations.^30^ Although these values do not admit biological explanations, they are considered to have meaningful effects in most comparisons. However, researchers must note that these are arbitrary values, and must use their judgment to assess whether these values are acceptable in their field of research.^24^, ^31^

## 3 SAMPLE SIZES FOR DESCRIPTIVE STUDIES

**FIGURE 2:**
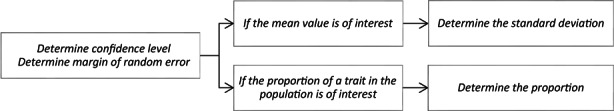
Elements of sample size calculation for descriptive studies.

A descriptive study is “concerned with and designed only to describe the existing distribution of variables, without regard to causal or other hypothesis.”^32^ Such studies include case reports, case series, and cross‐sectional (prevalence) studies.^33^ In the latter, the objective is to describe a health phenomenon in a population at a particular point in time. The main parameter of interest is proportion\prevalence, where the variable of interest is a categorical variable. In descriptive studies, the research could also be interested in the mean, where the variable of interest is a continuous response variable. For example, studies estimating the average age of children with asthma visiting the emergency room in a given year, or the prevalence of hyponatremia among the elderly in a tertiary care center, are descriptive in nature. The steps for sample size calculation in such descriptive studies are provided in Figure 2.

For example, a 95% confidence level indicates that the sample mean will not differ by more than a certain value from the true population mean in 95% of the repeatedly withdrawn samples from the same population. The margin of error (MoE) is a measure of the precision of an estimate. The smaller the allowed MoE, the larger the precision of our estimates and the larger the sample size. Note that the confidence interval = estimate of value of interest ± MoE. For example, if the prevalence of burnout is 15% in a sample of residents, then, for the larger population, it is estimated to be between 5% and 25% (allowing MoE of 10% on both sides). The standard deviation (SD) and the estimate of proportion can be obtained from previous studies. If no information regarding the SD is available, researchers can collect a pilot sample to estimate the value of SD, and use $\;\text{range}/\sqrt{n}$, where *n* is the number of observations in the pilot study.^34^ If that proportion is unknown, it is best to use a proportion close to what is expected; otherwise, a value of 0.5 is assumed to give a sufficiently large sample size.^35^ However, this value is appropriate if the actual population proportion is between 10% and 90%; otherwise (for example, in the case of rare or common disease), caution should be taken when substituting the proportion, as a significantly larger sample size is required.^36^

Note that population size is not needed as an input in most sample size calculations. The population can be defined by various elements, such as geographical, time frame, or social aspects. For example, if the prevalence of infection in a hospital's intensive care unit (ICU) department during the period between 2005 and 2012 is to be estimated, then the population is all the patients admitted to ICU during that period. In most studies, we aim to generalize the results to a larger population, although we are restricted to observing a specific population. Therefore, when estimating the sample size, population size is rarely important in medical research.^37^ However, if the population is limited (e.g., in a study that evaluates an academic program, where the population is all students enrolled in the program), then the sample size equations can be adjusted for the population size.^37^, ^38^, ^39^ The size of a finite population can be obtained from a database or records, or based on experience in the field, and is included in the sample size calculation.

## 4 SAMPLE SIZE FOR STUDIES COMPARING TWO GROUPS

There are two main types of study in health research: observational and experimental. An important distinction between the two is that, in an observational study, the researcher does not impose any intervention and observes only to assess a current condition. In experimental studies, an intervention is performed/conducted, and its results are observed. When the aim is to compare two groups (intervention/control), the number of study participants should be equally divided between both groups, so as to attain the maximum power for the given sample size. Note, however, that this point is limited to interventional studies and does not apply to observational studies (prospective vs. retrospective). The minimum sample size per group must be calculated based on the statistical test used. However, in some fields of study, such as pharmacology or biological research, a minimum of five per group is recommended and considered acceptable by academic journals in the field.^4^ Recommendations for minimum sample sizes for clinical studies suggest having at least 100 in each group.^40^ However, recent advances in sample size calculation have challenged these recommendations and have investigated the potential of simulation‐based methods.^41^, ^42^

Dividing participants equally between both groups might not be possible, for several reasons, e.g., costs or limited data on the treatment group in retrospective studies. In such cases, uneven groups are the best option at hand where the researcher will opt to increase the sample in one group (e.g., control) with available data.^43^ Attention should be paid to the statistical data analysis to be used^44^ and the method for reporting results. *p*‐values are generally large (above 0.05) in such cases,^45^ so reporting effect sizes^29^ and mean or median with confidence intervals can be more effective in conveying the practical importance of the results. All in all, increasing the sample size increases the precision of estimates, so it is important to report these measures.

## 5 PROBABILITY AND NONPROBABILITY SAMPLING

There are two types of sampling methods in research: probability (random) and nonprobability (nonrandom). In a probability sample, each unit has a known chance or probability of being selected. By contrast, in nonprobability sampling, units are withdrawn or chosen without specific probabilities. Probability sampling includes simple random sampling, systematic sampling, and stratified sampling. Nonprobability sampling includes convenience sampling and quota sampling.

Probability sampling has the advantages of higher generalizability, greater representativeness of the population, and lower response bias than nonprobability sampling.^46^ However, nonprobability sampling is the most commonly adopted type of sampling in clinical studies, survey statistics, and social research, due to its low‐ to no‐cost or for ethical reasons.^47^, ^48^, ^49^, ^50^ While calculating a sample size is important for the generalizability of results, estimating a sample size when using nonprobability sampling could be irrelevant, as convenience sampling is likely to generate nongeneralizable results, which preclude statistical inference to the larger population. As an alternative, researchers should include as many subjects as possible^51^ from the different subgroups and demographics. The quota sampling approach—or sample matching—might well be applied to minimize the selection bias often associated with nonprobability sampling.^52^ This is particularly useful if the hypothesis states that the main outcome of interest differs based on specific factors or exposure, such as gender or age group. The use of replication research studies to validate the results of nonprobability sampling is also encouraged as a strategy for ensuring generalizability.^53^ The methods section of a manuscript should include the number of subjects invited to participate or the size of target population (if known) and the number of participants instead of an actual sample size calculation.^49^ For a review on the inferential data analysis methods for nonprobability sampling, see Buelens, Burger, and van den Brakel,^46^ who applied machine learning methods in order to enhance the representativeness of the beforementioned sampling.

## 6 SAMPLE SIZE CALCULATION FOR REGRESSION ANALYSIS

Correlation or regression analysis is used in studies aiming to examine associations between a set of independent variables and a response variable. Failing to include an appropriate number of observations leads to an insufficient sample size, in which case regression might overfit the data.^54^ This means that, while the results may be valid for the study's dataset, they cannot be generalized to the population. In addition, estimates of regression coefficients are likely to be biased from true values, and the confidence intervals are large.^1^, ^11^ All these factors adversely affect statistical power. For regression analysis, several theories on sample size calculation have been provided in the literature regarding the use of logistic or linear regression for data fitting.^55^, ^56^, ^57^

The number of predictors is important for sample size calculation in regression analysis. A larger sample size is required for a higher number of predictors. In cases where interaction terms have more than two predictors, the number of interaction terms and the degree of interaction can become large. When the sample size is not large enough to conduct a similar regression analysis, one might add only important interaction terms with a large effect or use practical judgment to form the interaction terms.

Another important element in sample size calculation is the *R*‐squared, defined as the measure of the strength of association between the regression model and the response; it is also defined as the proportion of the variance in the response that is explained collectively by the independent variables.^58^ Calculating the sample size required for multiple regression analysis is equivalent to ascertaining the number of subjects to be enrolled to produce an acceptable *R*‐squared or goodness‐of‐fit. Multiple regression analysis aims to determine whether a variable is significantly associated with the outcome after controlling for all the other predictors. For purposes of estimating the effect size in multiple regressions of each variable, an assumption is made regarding the value of the *R*‐squared, because the exact estimates of regression coefficients of these variables are unknown. It is then possible to calculate Cohen's *f* ^2^ effect size, which is defined as the ratio of the proportion of variance accounted for relative to the proportion of a variable unaccounted for, where *f* ^2^ is classified as small, medium, or large (*f* ^2^ = 0.02, 0.15 or 0.35, respectively) effect sizes.^29^

Calculating sample size on the assumption that regression analysis is to be used is not practical in many cases. For example, in any study, there may be more than one multiple regression model, and estimating the sample size for each model is not practical. Although it is common practice to estimate a sample size sufficient to estimate the minimum effect size, a minimum effect size might not be identifiable in some cases. Hence, researchers have often relied on “rules‐of‐thumb” to determine approximate sample sizes. For example, one of the considered rules‐of‐thumb calls for 10 observations per variable.^59^ In addition, the sample size should be larger than the number of predictors, or else the regression coefficient cannot be estimated. How much larger the sample size needs to be is an issue of debate and depends on the field of study, e.g., biological or social research. Green^60^ challenges most of the commonly used rules and argues for an approach that considers the effect sizes. While he has provided some support for the latter, he also argues that it is not appropriate when dealing with seven or more model predictors, though it is suitable when there is a medium‐sized association between the response and predictors. More recent proposals in sample size determination reportedly overcome the design or practical challenges in the field.^7^, ^59^

## 7 GENERAL RECOMMENDATIONS

### 7.1 Account for nonresponsiveness

Researchers must face the reality that not all invited participants are willing to be enrolled, which entails the possibility of a low response rate. A large difference between the calculated sample size and actual number of subjects in a study affects the generalizability of the results. Furthermore, the sample collected might not be large enough for the planned statistical analysis. Researchers often predict the response rate. For example, in clinical research, the dropout or noncompliance rate is around 10%.^61^ Accounting for dropout or nonresponsiveness is particularly important in many studies, such as longitudinal studies requiring follow‐up trials/studies.^62^

Suppose *n* participants are required in a study, but a percentage (*p*) are projected to dropout or are nonresponsive. In such a scenario, more subjects must be approached in order to achieve the planned sample size. Hence, the edited final sample size is

$$n\left(\text{final}\right)=\frac{n}{\left(1-p\right)}.$$

The value of the response rate is often derived from experience or previous research. For example, to estimate the proportion of burnout in staff residents in a regional hospital, consider a sample with 15% burnout. Allowing for an MoE of 5% and a confidence level of 95%, the minimum sample size is 195.9. The recommended sample size can be set at 245, so as to allow for a 20% nonresponse rate. Note that a large nonresponse rate is assumed here, as the population involves physicians.^63^

### 7.2 Avoid unrealistically large samples

For start‐up studies or studies where no previously established literature is available, we recommend opting for medium to large effect sizes and not setting sample sizes based on the minimum effect that would be of practical significance. The results of such studies can provide insights and useful information for future meta‐analyses. This also applies if the research is an undergraduate project with limited resources.

For example, a researcher comparing the incidence of a certain outcome between two independent groups might initially be interested in serious complications in patients exposed to two distinct surgical treatments. However, if this number is very small, a large sample size will be required. If resources allow, the researcher should perhaps investigate whether there is a sufficiently large number of surgeries in the current hospital; if there is not, it may be advisable to cover more centers. Alternatively, these researchers could alter their research question so that it is concerned with the incidence of any complications following the procedure, and not limited to serious complications. Hence, the required sample size would be smaller and more feasible. In short, researchers should always look at the sample size and judge whether it is reasonable and suited to their research question(s).

## 8 SUMMARY

Sample size calculation is the principal component of a quantitative study. Ethical committees consider it a prerequisite for the approval of a research study. However, sample size calculation is challenging and often relies on certain assumptions, which are rarely accurate. Determining the required sample size should not be considered an answer to a research question. The final decision should be guided by cost and time limitations, as well as clinical or practical judgment.

## AUTHOR CONTRIBUTIONS

**Alaa Althubaiti** involved in conceptualization, investigation, formal analysis, methodology, project administration, writing—original draft, and writing—review and editing.

## ETHICAL APPROVAL

Ethical approval was waived by the Ethics Committee in view of the study type.

## PATIENT CONSENT STATEMENT

None.

## CLINICAL TRIAL REGISTRATION

None.

## CONFLICT OF INTEREST

The authors have stated explicitly that there are no conflicts of interest in connection with this article.

## Supporting information


Table S1

## Data Availability

Data sharing not applicable—no new data generated.
