# Supplementary material for: Sample size determination: A practical guide for health researchers
Source: J Gen Fam Med. 2022 Dec 14;24(2):72–8. doi: 10.1002/jgf2.600 (PMC10000262; doi:10.1002/jgf2.600)
Supplement: Supplementary file 1 — Table S1 [file JGF2-24-72-s001.docx]

**Supplement 1**. Statistical terms for sample size calculation

|  | **Definition** |
| --- | --- |
| **Hypothesis testing** | - The hypothesis is a statement the researcher formulates related to the population value of interest. - In the process of hypothesis testing, the researcher establishes a null and alternative hypothesis, withdraw a sample to examine the hypotheses, and decide on the statistical test to be used. |
| **Two-sided test and one sided and** | - In one sided test, the aim of study is to test whether a value of interest (e.g., mean or proportion) is being less than or greater than a prespecified value. In two-sided test, the test investigates if the value of interest differs in any direction from a predefined value or an estimate. |
| **Power** | - It is an arbitrary probability value for the act of correctly rejecting a false null hypothesis. |
| **Level of confidence** | - Probability that an estimate of a population parameter is within certain specified limits of the true value |
| **Type I or false positive** | - Rejecting the null hypothesis when it is true |
| **Type II or false negative** | - Failing to reject the null hypothesis when it is false. Power calculations provide information on how many participants are required to avoid a type II error. |
| **α** | - The probability of making type I error. The significance level determined at the start of the study. α =0.05 is the common level used in medical, biological, social and educational research. The size of power depends on α. |
| **β** | - The probability of making type II error. |
